# Diagnosis of vascular calcification related to mineral and bone metabolism disorders in chronic kidney disease

**DOI:** 10.1590/2175-8239-JBN-2021-S104

**Published:** 2021-12-03

**Authors:** Fabiana Oliveira Bastos Bonato, Cristina Karohl, Maria Eugênia Fernandes Canziani

**Affiliations:** 1 Universidade Federal de Juiz de Fora, Juiz de Fora, MG, Brazil.; 2 Universidade Federal do Rio Grande do Sul, Porto Alegre, RS, Brazil.; 3 Universidade Federal de São Paulo, Nephrology Discipline, São Paulo, SP, Brazil.

**1.** The presence of valve and vascular calcification (VC) should be investigated annually in patients with CKD G3a-5D by means of radiological examinations (lateral abdominal or pelvic and hand radiographs) and echocardiography, respectively (Opinion).

**2.** CKD G3a-5D patients with known vascular and/or valve calcification should be considered at high cardiovascular risk (Evidence), and this information is valid to guide the treatment of CKD-MBD (Opinion). 

## Rational

Cardiovascular diseases (CVD) are the leading cause of mortality in patients with chronic kidney disease (CKD)^1^. Valve and vascular calcifications (VC) are among the most frequent complications of CKD, being one of the components of mineral and bone disorder in CKD (CKD-MBD)^2^. The prevalence of VC increases with CKD progression, and may be present in more than 30% of patients under conservative treatment^3^ ^,^ ^4^ and in more than 80% of those on dialysis^5^ ^,^ ^6^. Valve calcification is also common and it is observed in about 20% to 25% of CKD patients undergoing conservative treatment^7^ and between 32% to 76% of patients on dialysis^8^ ^,^ ^9^. 

There are several risk factors for VC development and progression in CKD, including traditional risk factors such as advanced age, presence of diabetes *mellitus*, systemic arterial hypertension, dyslipidemia, smoking, and obesity, and risk factors associated with the kidney disease itself. VC could be present in both the intima and media layers of arteries^10^. The calcification located in the intima of the vessel is typically secondary to systemic inflammation and established atherosclerosis^11^. CKD patients, especially those on dialysis, have a chronic inflammatory state that is an important contributor to endothelial cell damage and accelerated progression of atherosclerotic plaque^10^ ^,^ ^12^. On the other hand, VC located in the media layer of the vessel is associated with CKD-MBD, being an active process of vessel "ossification" linked to an imbalance between inhibitor and promoter factors of calcification^10^ ^,^ ^13^ ^,^ ^14^ (Table 1). In the presence of calcium (Ca), phosphorus (P) and uremic toxins overload, the vascular smooth muscle cells undergo a phenotypic transformation and acquire characteristics of osteoblastic cells, producing bone matrix inside the vascular wall^10^ ^,^ ^15^. A study demonstrates that elevated PTH levels may also play a role in the pathophysiology of VC^16^. Valve calcification in CKD patients, in turn, is also related to CKD-MBD, inflammation, oxidative stress, and pressure and volume overload^17^. 

**Table 1.** Promoter and Inhibitor Factors of Vascular Calcification^14^

Promoters	Inhibitors
BMP-2, 4 and 6	Matrix Gla Protein
Osteocalcin	Osteopontin
Bone Sialoprotein	Osteoprotegerin
Alkaline phosphatase	Fetuin A
Calcium and phosphorus	Klotho
Oxidative Stress	Pyrophosphate
Inflammatory cytokines	Carbonic anhydrase
Diabetes	BMP-7
Dyslipidemia	Vitamin K
Coumarins	Magnesium
Matrix exosomes	Sodium thiosulfate
Apoptotic cells	
MMP2, 3 and 7	
Runx2	
Sox9	
Osterix/Sp7	

BMP: Bone morphogenetic protein; MMP: Matrix metalloproteinases; Runx2, Sox9 and Osterix: Osteochondrogenic transcription factors.

Defining the exact prognostic value of VC is complex in CKD patients due to the difficulty in discriminating the histological type, whether in the intima or media layer, and the pathophysiological mechanisms of calcification, which are distinct in relation to the general population^18^ ^,^ ^19^. However, the loss of vessel elasticity, myocardial ischemia and fibrosis caused by VC in CKD lead to increased mortality and higher risk of cardiovascular events^10^ ^,^ ^20^ ^,^ ^21^. Valve calcification, on the other hand, contributes to increased afterload, worsening left ventricular hypertrophy and myocardial fiber disarray^14^. The end product of vascular, valve, and myocardial changes is increased risk of arrhythmias, heart failure, and sudden death^14^ ^,^ ^22^ ^,^ ^23^. The absence of VC, in general, is associated with good prognosis^24^. 

Although, to date, there is no specific therapy capable of regressing calcification, an annual investigation is recommended in order to identify patients at increased cardiovascular risk. Diagnosis of VC and valve calcification should be easy to perform and interpret, accurate, reproducible, safe, and cost-effective. Several methods have been used to detect and quantify valve and VC, but none of them could be considered optimal. In addition, methods to assess VC are usually not able to differentiate its location, whether in the intima or the media layer^10^. The currently available methods could be classified as qualitative, semiquantitative and quantitative. 

## A. Qualitative

**A.1** Plain radiography: accessible method, as it is easy to obtain and has a low cost, which allows detecting the presence of VC in vessels of different body segments. However, it has low sensitivity and it is unable of quantifying the severity of VC^25^.

**A.2** Arterial ultrasound: qualitative method that allows the assessment of VC presence in common carotid arteries, abdominal aorta and iliac-femoral arteries. Although it is a safe and relatively low-cost method, ultrasound has the disadvantage of being operator dependent. The presence of VC assessed by this method was associated with cardiovascular mortality in CKD patients^24^ ^,^ ^26^. Furthermore, one study suggested that measuring the carotid arteries intima-media thickness could identify not only the presence but also the progression of VC^27^. However, studies are still limited in this population. 

**A.3** Echocardiogram: safe, reliable, and accessible exam capable of detecting calcification and mitral and aortic valve stenosis, with the additional benefit of early diagnosis of changes in cardiac function. It has the advantage of not requiring the use of iodinated or gadolinium contrast, which should be avoided in the CKD population^17^. 

## B. Semiquantitative

**B.1** Lateral lumbar spine radiography: the best semiquantitative method was described by Kauppila et al. based on calcification foci number and extent in the aortic segments at the level of the first to the fourth lumbar vertebrae, applying a score from 0-24^28^. This score showed good correlation with coronary calcification detected by computed tomography and it was also associated with mortality and cardiovascular events in dialysis patients^29^ ^,^ ^30^. The use of this tool could help in the risk stratification of this population. 

**B.2** Hand and pelvis radiographs: simple, available, low-cost, and easy-to-interpret method for VC assessment; it consists of dividing hand and pelvis radiographs into quadrants. The final score is the sum of the quadrants with calcification, ranging from 0 to 8^31^. This method showed a significant correlation with coronary calcification and mortality in CKD patients^32^. 

## C. Quantitative

**C.1** Coronary electron-beam computed tomography or multislice computed tomography: non-invasive techniques with low radiation exposure and no need to use contrast, being considered the gold standard for presence and quantification of VC. When performed in different periods, they allow the analysis of calcification progression^24^. Presence of calcium in the coronary artery is quantified by the Agatston score, which is calculated by multiplying the plaque area by a density coefficient (measured in Hounsfield units) and the result is given by summing the score of each coronary lesion. This method does not allow the distinction between calcification of the intima and media layers^33^. Studies show that coronary tomography has a prognostic role, since the presence and progression of VC are associated with cardiovascular complications and all-cause mortality in hemodialysis patients^10^ ^,^ ^34^ ^,^ ^35^.

It is important to note that, to date, there is no specific therapy for VC regression and it is unclear how reducing its speed of progression impacts the outcomes of CKD patients. The recommendation to perform annual plain abdominal radiography to detect VC and echocardiogram to detect valve calcification and cardiac functional assessment has as main objectives assessing the mortality risk and implementing measures that may delay the progression of VC. In this sense, the positivity of any of these tests points to an increased cardiovascular risk of the patient, impacting on the individualization and intensification of the management of modifiable risk factors^2^ ^,^ ^21^. As for the calcification quantification, it is not usually performed routinely, since CT scans are expensive and expose the patient to radiation. However, it can be considered in cases of positive qualitative tests, with the purpose of better prognostic stratification, especially in situations of research^10^ ^,^ ^14^.
